# Interactions of blood biomolecules with early rhythm control in atrial fibrillation patients: exploratory analysis of the EAST-AFNET 4 biomolecule study

**DOI:** 10.1093/europace/euag149

**Published:** 2026-06-23

**Authors:** Christoph Al-Taie, Julius Obergassel, Katrin Borof, Julius Ridder, Andreas Rillig, Andreas Metzner, Andreas Goette, Christina Magnussen, Moritz F Sinner, Laura C Sommerfeld, Stephan Willems, Tanja Zeller, Renate B Schnabel, Ulrich Schotten, Antonia Zapf, Paulus Kirchhof, Larissa Fabritz

**Affiliations:** University Centre of Cardiovascular Science, University Medical Center Hamburg Eppendorf, Martinistr 52, 20246 Hamburg, Germany; Department of Cardiology, University Heart and Vascular Centre Hamburg (UHZ), Martinistr. 52, 20246 Hamburg, Germany; German Centre for Cardiovascular Research (DZHK), Partner Site North, Martinistr. 52, 20246 Hamburg, Germany; University Centre of Cardiovascular Science, University Medical Center Hamburg Eppendorf, Martinistr 52, 20246 Hamburg, Germany; Department of Cardiology, University Heart and Vascular Centre Hamburg (UHZ), Martinistr. 52, 20246 Hamburg, Germany; German Centre for Cardiovascular Research (DZHK), Partner Site North, Martinistr. 52, 20246 Hamburg, Germany; Department of Cardiology, University Heart and Vascular Centre Hamburg (UHZ), Martinistr. 52, 20246 Hamburg, Germany; University Centre of Cardiovascular Science, University Medical Center Hamburg Eppendorf, Martinistr 52, 20246 Hamburg, Germany; Department of Cardiology, University Heart and Vascular Centre Hamburg (UHZ), Martinistr. 52, 20246 Hamburg, Germany; German Centre for Cardiovascular Research (DZHK), Partner Site North, Martinistr. 52, 20246 Hamburg, Germany; Department of Cardiology, University Heart and Vascular Centre Hamburg (UHZ), Martinistr. 52, 20246 Hamburg, Germany; German Centre for Cardiovascular Research (DZHK), Partner Site North, Martinistr. 52, 20246 Hamburg, Germany; Department of Cardiology, University Heart and Vascular Centre Hamburg (UHZ), Martinistr. 52, 20246 Hamburg, Germany; German Centre for Cardiovascular Research (DZHK), Partner Site North, Martinistr. 52, 20246 Hamburg, Germany; Department of Cardiology and Intensive Care Medicine, Saint Vincenz Hospital Paderborn, Paderborn, Germany; Medical Faculty, Otto-von-Guericke University, Magdeburg, Germany; Atrial Fibrillation NETwork (AFNET), Mendelstr. 11, 48149 Münster, Germany; Department of Cardiology, University Heart and Vascular Centre Hamburg (UHZ), Martinistr. 52, 20246 Hamburg, Germany; German Centre for Cardiovascular Research (DZHK), Partner Site North, Martinistr. 52, 20246 Hamburg, Germany; Atrial Fibrillation NETwork (AFNET), Mendelstr. 11, 48149 Münster, Germany; Department of Medicine I, LMU University Hospital of Munich, Munich, Germany; German Centre for Cardiovascular Research (DZHK), Partner Site Munich Heart Alliance, Munich, Germany; University Centre of Cardiovascular Science, University Medical Center Hamburg Eppendorf, Martinistr 52, 20246 Hamburg, Germany; Department of Cardiology, University Heart and Vascular Centre Hamburg (UHZ), Martinistr. 52, 20246 Hamburg, Germany; German Centre for Cardiovascular Research (DZHK), Partner Site North, Martinistr. 52, 20246 Hamburg, Germany; Atrial Fibrillation NETwork (AFNET), Mendelstr. 11, 48149 Münster, Germany; Asklepios Clinic St.Georg, Cardiology and Internal Intensive Care Medicine, Hamburg, Germany; German Centre for Cardiovascular Research (DZHK), Partner Site North, Martinistr. 52, 20246 Hamburg, Germany; Institute for Cardiogenetics, UKSH, Lübeck, Germany; University Centre of Cardiovascular Science, University Medical Center Hamburg Eppendorf, Martinistr 52, 20246 Hamburg, Germany; Department of Cardiology, University Heart and Vascular Centre Hamburg (UHZ), Martinistr. 52, 20246 Hamburg, Germany; German Centre for Cardiovascular Research (DZHK), Partner Site North, Martinistr. 52, 20246 Hamburg, Germany; Atrial Fibrillation NETwork (AFNET), Mendelstr. 11, 48149 Münster, Germany; Atrial Fibrillation NETwork (AFNET), Mendelstr. 11, 48149 Münster, Germany; Department of Physiology, Maastricht University, Maastricht, The Netherlands; German Centre for Cardiovascular Research (DZHK), Partner Site North, Martinistr. 52, 20246 Hamburg, Germany; Atrial Fibrillation NETwork (AFNET), Mendelstr. 11, 48149 Münster, Germany; Institute of Medical Biometry and Epidemiology, University Medical Center Hamburg Eppendorf, Hamburg, Germany; Department of Cardiology, University Heart and Vascular Centre Hamburg (UHZ), Martinistr. 52, 20246 Hamburg, Germany; German Centre for Cardiovascular Research (DZHK), Partner Site North, Martinistr. 52, 20246 Hamburg, Germany; Atrial Fibrillation NETwork (AFNET), Mendelstr. 11, 48149 Münster, Germany; Cardiovascular Sciences, University of Birmingham, Birmingham B15 2TT, UK; University Centre of Cardiovascular Science, University Medical Center Hamburg Eppendorf, Martinistr 52, 20246 Hamburg, Germany; Department of Cardiology, University Heart and Vascular Centre Hamburg (UHZ), Martinistr. 52, 20246 Hamburg, Germany; German Centre for Cardiovascular Research (DZHK), Partner Site North, Martinistr. 52, 20246 Hamburg, Germany; Atrial Fibrillation NETwork (AFNET), Mendelstr. 11, 48149 Münster, Germany; Cardiovascular Sciences, University of Birmingham, Birmingham B15 2TT, UK

**Keywords:** Atrial fibrillation, Early rhythm control, Cardiovascular biomarkers, BMP10, Interaction analysis

## Abstract

**Aims:**

Early rhythm control reduces cardiovascular events in patients with atrial fibrillation (AF) and cardiovascular comorbidities. Whether this effect is consistent across severities of different AF-associated conditions is not known.

**Methods:**

In the EAST-AFNET 4 biomolecule study (*n* = 1586; median age 70 years; 45% women), 14 circulating biomarkers reflecting inflammation, fibrosis, ageing, cardiac strain, and myocardial damage were quantified. Log-transformed, winsorized biomarkers were analysed in quintiles to detect non-linear associations and as continuous parameters. Cox proportional hazards models evaluated associations with the primary outcome (cardiovascular death, stroke, or unplanned hospitalization for heart failure or acute coronary syndrome) and the safety outcome (death, stroke, or major bleeding) of the trial, including interaction terms for biomarkers and randomized treatment [early rhythm control (ERC) vs. usual care (UC)]. Two independent cohorts, BBC-AF resembling UC and a TRUST snapshot resembling ERC were combined for external replication. All tests were exploratory.

**Results:**

ERC reduced the primary outcome consistently across biomarker concentrations. No signal for treatment interaction was observed for 13 of 14 biomarkers for the primary outcome. For BMP10, a nominal interaction was observed in categorical analyses, suggesting an attenuated treatment effect of ERC in patients in the lowest BMP10 quintile. No interaction was detected for continuous parameters. Similar patterns were observed in exploratory analyses of the replication cohort.

**Conclusion:**

ERC therapy is effective across patients with different severities of conditions associated with AF as quantified by biomolecule concentrations. A potentially attenuated effect of ERC with low BMP10 concentrations warrants further research.

This analysis is part of the EAST-AFNET 4 trial biomolecule study, the original trial has been registered under the official title: Early Therapy of Atrial Fibrillation for Stroke Prevention Trial (EAST) (EudraCT 2010-021258-20) at https://clinicaltrials.gov/study/NCT01288352.

## Graphical Abstract

**Graphical Abstract:**
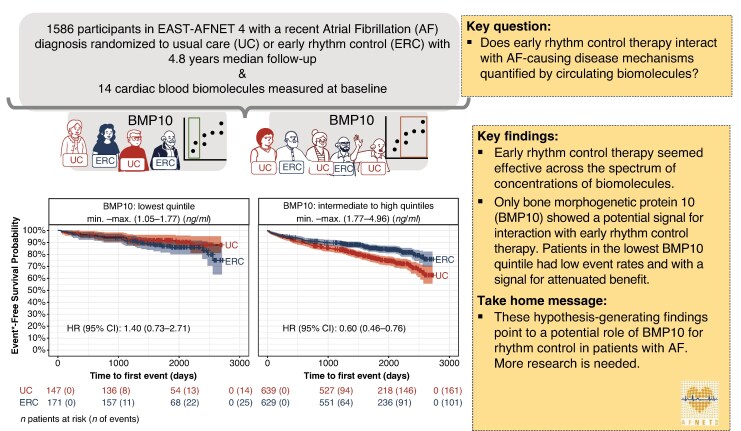
Grouping according to BMP10 measured at baseline stratifies the patients into distinct risk groups in which the treatment effect of early treatment is being moderated. The signal for a possible interaction effect between ERC treatment group and BMP10 in association with the primary outcome persists and strengthens when participants are dichotomized into two BMP10 concentration groups build on quintiles.

What’s new?

- In this exploratory analysis, concentrations of 13 of 14 biomolecules, including biomolecules reflecting cardiac strain and damage (NT-proBNP, high-sensitivity troponin), inflammation and ageing (CRP, IL-6, GDF-15), and fibrosis (FGF23) did not show evidence of interactions with early rhythm control therapy in patients with atrial fibrillation, supporting a broadly consistent treatment effect across the severity of conditions associated with AF.
- For the novel biomarker bone morphogenetic protein 10 (BMP10), a nominal interaction signal was observed in categorical analyses, suggesting the possibility of an attenuated effect of early rhythm control in patients with low BMP10 concentrations, while no interaction was detected in continuous models. This hypothesis-generating signal warrants further research.

## Introduction

Early rhythm control (ERC) prevents cardiovascular death, stroke, and hospitalization due to worsening of heart failure or acute coronary syndrome^1,2^ in patients with recently diagnosed atrial fibrillation (AF) and stroke risk factors in the EAST-AFNET 4 trial. ERC was delivered using antiarrhythmic drugs^3,4^ or AF ablation.^1^ In subgroup interrogations of the trial,^5–12^ the only feature that interacted with the effect of ERC therapy was a high comorbidity burden.^13^ To probe the influence of different disease mechanisms related to AF in the EAST-AFNET 4 trial, preselected biomolecules reflecting different metabolic and cellular disease mechanisms were quantified in the EAST-AFNET 4 biomolecule study. Quantified biomarkers reflect inflammation [C-reactive protein (CRP), interleukin-6 (IL-6)], thrombosis (D-Dimer), vascular dysfunction [angiopoietin 2 (ANGPT2)], cardiac damage [troponin (TnT)], cardiac and atrial strain [*N*-terminal pro b-type natriuretic peptide (NT-proBNP)], autonomic dysfunction and ageing [growth differentiation factor 15 (GDF-15), insulin-like growth factor binding protein 7 (IGFBP7)], fibrosis [fibroblast growth factor 23 (FGF23)], atrial metabolic stress [bone morphogenetic protein 10 (BMP10)] and kidney function (serum creatinine sCr).^14^ Patient clusters identified by unsupervised analyses of these biomolecules define four clusters of patients with distinct risk of AF-related complications^15^ and of recurrent AF.^16^ To explore whether these biomolecules identify subgroups of patients with different severity of conditions associated with AF who respond differently to ERC, this hypothesis-generating analysis determined interactions of baseline concentration of each biomolecule with the ERC therapy treatment group in the EAST-AFNET 4 biomolecule study.

## Methods

### EAST-AFNET 4 biomolecule study and biomarker quantification

**Figure 1:**
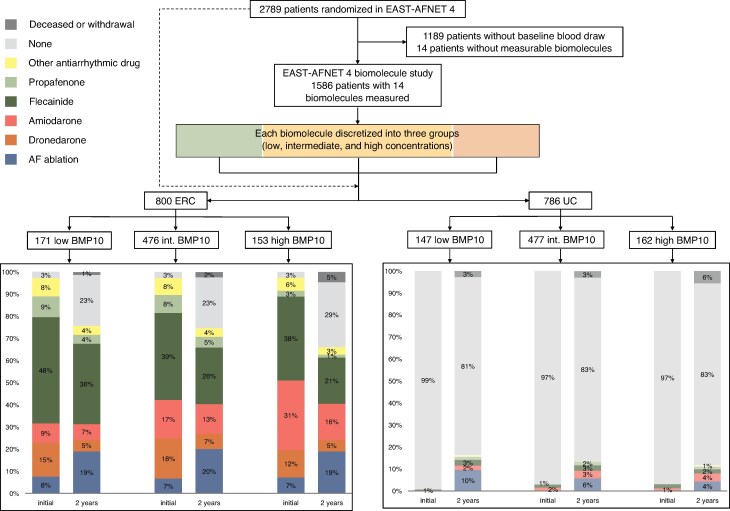
CONSORT diagram of this analysis exemplified for discretized BMP10. Path of patients from enrolment to groups. First lead by randomization of the main RCT followed by patients’ assignment to discrete groups lead by quantification of BMP10 concentrations. ERC, early rhythm control; int., intermediate; UC, usual care.

This is an exploratory analysis of the EAST-AFNET 4 biomolecule study that includes 1586/2789 patients randomized to ERC or usual care in the EAST-AFNET 4 trial (*Figure 1*).^1,15,17^ Samples were collected at baseline and shipped to the central bio-storage facility at UKE Hamburg at ambient temperature, where they were centrifuged, shock-frozen, and stored at −80°C until analysis. The EAST-AFNET 4 study and its biomolecule study received approval at all study sites. Written informed consent was obtained from all participants. Absolute protein concentrations were centrally quantified in EDTA plasma, with rigorous quality controls using high-throughput, high-precision pre-commercial analysers (Roche, Penzberg, Germany) as an in-kind contribution of Roche to the Horizon2020 CATCH ME EU-funded consortium.^14^ Blood samples were shipped to the Roche biomarker research facility in Penzberg, Germany. Biomarkers were measured using high throughput, high-precision electrochemiluminescence immunoassays on cobas® e601 and photometric assays on cobas® c501 analysers (Roche Diagnostics, Penzberg, Germany). All assays demonstrated intra- and inter-assay coefficients of variation generally below 10% across clinically relevant concentration ranges. For example, the Elecsys NT-proBNP assay demonstrates total coefficients of variation of approximately 2.9–6.1% across clinically relevant concentrations.^18^ The BMP10 assay shows coefficients of variation of about 4.3–6.0% for concentrations of 3.56 and 1.39 ng/mL, respectively.^19^ Find the Spearman correlation for all biomolecules in Supplementary material online, *Figure S1*.

### Exploratory replication

The EAST-AFNET 4 biomolecule dataset is quite unique, as it stems from a randomized controlled trial of ERC and contains biomarker concentrations, including novel preclinical biomarkers. For exploratory replication in independent observational cohorts, data from 801 AF patients of the BBC-AF registry^20^ and 651 patients from a TRUST^21^ snapshot with AF diagnosed less than 1 year prior to enrolment were combined. BBC-AF patients rarely received rhythm control therapy, rendering them comparable to usual care in EAST-AFNET 4. The TRUST patients almost universally underwent AF ablation and therapy with antiarrhythmic drugs, resembling ERC. Blood biomarkers (ANGPT2, BMP10, FGF23, IGFBP7, and NT-proBNP) were quantified using the same assays as in the EAST-AFNET 4 biomolecule study.

### Preprocessing of biomolecule concentrations

**Figure 2:**
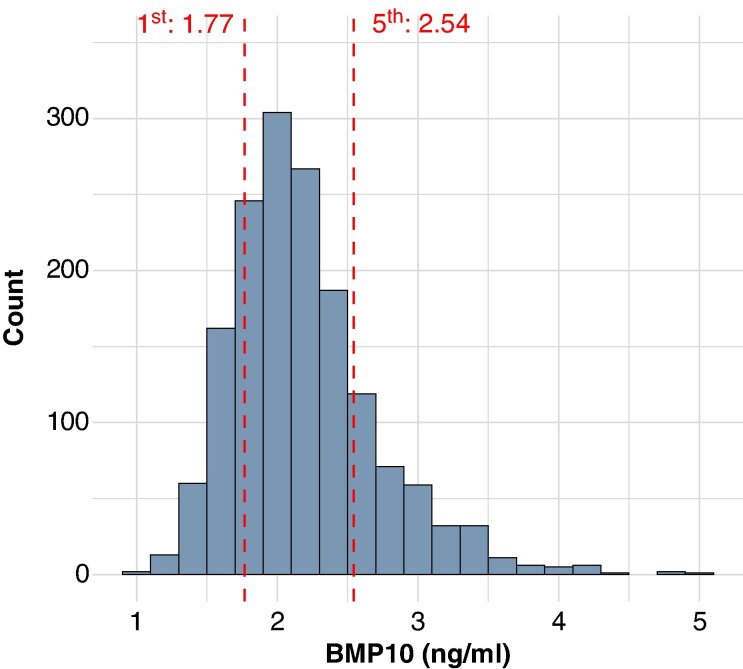
Distribution of bone morphogenetic protein 10 (BMP10) blood plasma concentrations in EAST-AFNET 4 patients with discretization boundaries—1st and 5th quintile.

The EAST-AFNET 4 biomolecule study analysis was prespecified in a statistical analysis plan (see Supplementary data). Only patients with complete data were included in this analysis. To bring the quantified biomolecule values into an approximate normal distributed shape and to mitigate the effects of outliers, we applied natural log-transformation and 1% upper winsorization.^22^ To detect non-linear effects, we discretized each continuously scaled biomolecule into an ordinally scaled variable. Therefore, we used the 20th percentile as lower- and the 80th percentile as upper bound to categorize into low, medium, and high biomolecule concentrations, employing a common approach for assessing the impact of polygenic risk scores (*Figure 2*).^23,24^

### Statistical analysis

The primary efficacy outcome was a composite of cardiovascular death, stroke, or unplanned hospitalization for heart failure or acute coronary syndrome. The safety outcome is a composite of death from any cause, stroke, or serious adverse event of special interest related to rhythm-control therapy (SAE).^1^ Both outcomes are the main outcomes in EAST-AFNET 4. Each biomolecule was used to construct a Cox proportional hazard (Cox PH) model^25^ with the primary outcome as time-to-event endpoint, the study centre as frailty term and an interaction term between the biomarker concentration and the randomized group. All models were adjusted for sex, age, and sinus rhythm at blood draw. We contrasted each model with a nested model without the interaction term and compared both models’ goodness of fit by ANOVA. With a confidence level of 95%, we indicate suggestion of heterogeneity [effect modification through a (non-linear) relationship] across biomarker levels on the randomized treatment in EAST-AFNET 4. Hazard ratios (HRs) and *P*-values for the treatment effects under different discretized biomarker levels were calculated by reference cell coding.^26^ When a *P*-value for interaction passed the threshold of 0.05, we applied the same statistical models to the components of the primary outcome for this biomolecule. For sensitivity analysis we plotted the event-free survival probability for both treatment groups stratified by discretized biomarker-defined patient groups. Biomarkers were analysed in quintiles to explore potential non-linear associations and to facilitate clinical interpretability. All analyses are hypothesis-generating. Following a recommendation from the European Medicines Agency on secondary analyses of controlled clinical trials, *P*-values were not corrected for multiple testing. To describe the false positive rate, we calculated corrected *P*-values (*q*-values)^27^ for the first primary outcome in EAST-AFNET 4. The results mainly serve to generate signals for further investigation.^28^ All analysis were conducted using R^29^ version 4.2.2. Details of all used packages are shown in Supplementary material online, *Table S1*.

## Results

**Figure 3:**
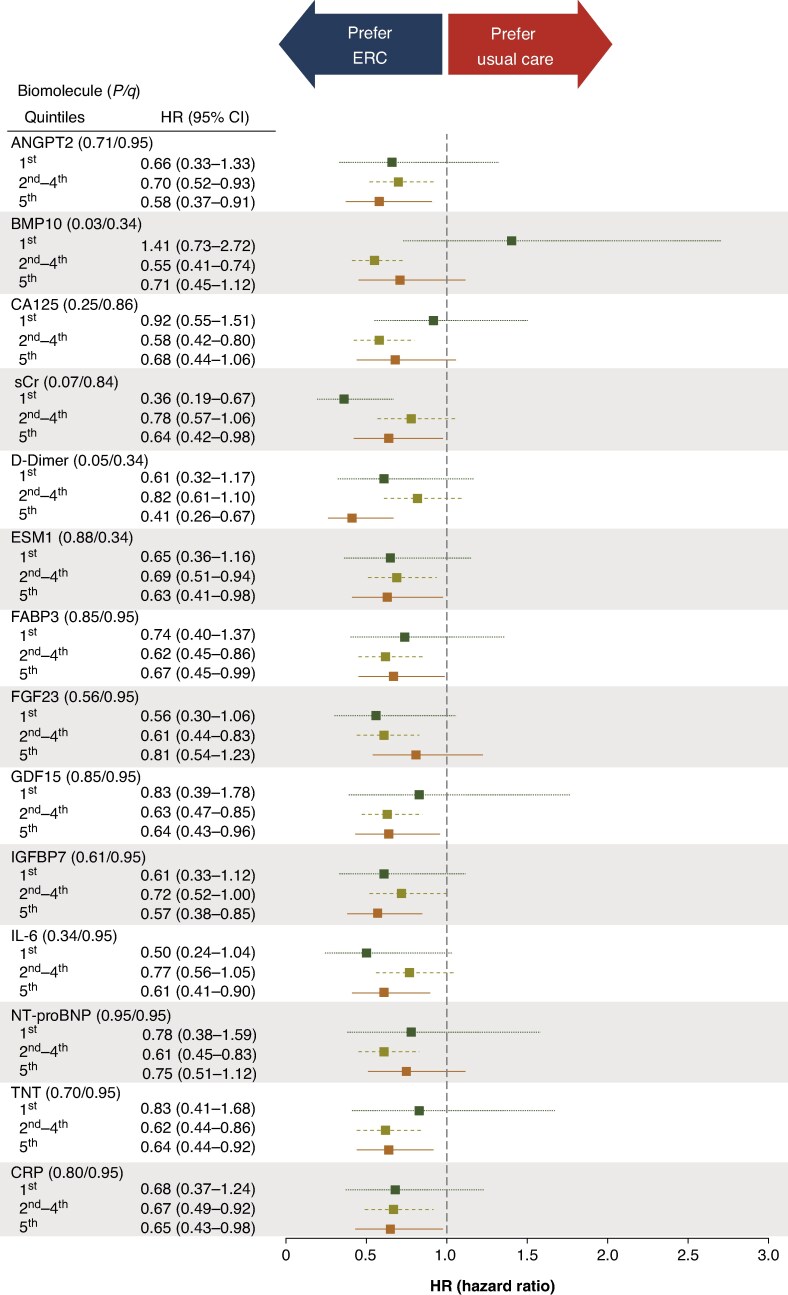
Biomolecule interaction analysis with early rhythm control therapy. HRs of early rhythm control [vs. usual care stemming from Cox proportional hazard (PH) models showing HRs alongside 95% CIs for the treatment] in interaction with each low, intermediate and high discretized biomolecule concentration group. *P*-values for interaction were calculated using ANOVA comparing each nested Cox PH model pair. The *q*-values for interaction were calculated applying false discovery rate (FDR). BMP10 shows a nominal interaction signal with early rhythm control therapy.

**Figure 4:**
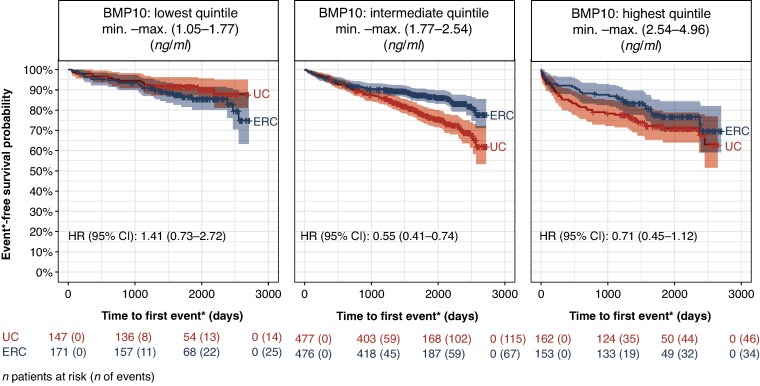
Primary outcome. Interaction analysis of BMP10 (low, intermediate, and high BMP10 concentrations) with early rhythm control group. Estimated event-free survival probability for the *first primary outcome stratified by discretized BMP10 over the follow-up time. Hazard ratios stem from Cox proportional hazard models.

Clinical features of the EAST-AFNET 4 biomolecule study and distributions of biomolecule concentrations have been reported (see also *Tables 1* and *2*).^12,13^ Concentration boundaries for the biomolecules are given in *Table 1*. When patients were grouped into low, intermediate, and high biomolecule concentrations (*Figure 1*), there was no signal for an interaction with ERC for 13 of the 14 biomolecules (*Figure 3*). Only the discretized BMP10-based groups showed a nominal interaction signal with ERC therapy that would not persist after adjustment for multiple testing (*P* = 0.032, *q* = 0.34): In patient with low BMP10 concentrations, Aalen–Johansen survival curves for the primary outcome (time to cardiovascular death, stroke, or unplanned hospitalization for heart failure or acute coronary syndrome) show fewer patients with events when receiving UC (*n* = 14) than with ERC (*n* = 25), patients with intermediate BMP10 concentrations: UC *n* = 115, ERC *n* = 67 and with high BMP10 levels: UC *n* = 46, ERC *n* = 34 (*Figure 4*). The number of patients with event in each group is given in *Table 3*. Patients with low BMP10 concentrations are characterized by younger age, higher likelyhood of being in sinus rhythm at baseline, less persistent AF, and male sex (*Table 2*). None of these features interacts with ERC therapy.^11,30^ Low BMP10 concentrations identify a group of patients at low risk of cardiovascular events (*Figure 4*) and potentially with attenuated benefit of ERC. Event numbers and rhythm at 12- and 24-month follow-up are indicated in *Table 3*. For each component of the primary outcome, the absolute number of events is lower in patients assigned to UC than in patients assigned to ERC (*Table 3*) for patients in the lowest quintile of BMP10 concentrations.

**Table 1:** Blood biomolecule concentrations by discretized concentrations in three groups according to quintiles

		Median [IQR] [range]		
Biomolecule	Unit	Low 1st quintile	Intermediate 2nd – 4th quintile	High 5th quintile
**ANGPT2**	ng/ml	1.5 [1.3–1.6] (0.22–1.72)	2.6 [2.1–3.2] (1.72–4.24)	5.90 [5.0–7.8] (4.24–26.36)
**BMP10**	ng/ml	1.6 [1.5–1.7] (1.05–1.77)	2.1 [2.0–2.3] (1.77–2.54)	2.9 [2.7–3.2] (2.54–4.96)
**CA125**	U/ml	6.0 [5.0–6.8] (2.03–7.40)	11.4 [9.5–13.6] (7.41–17.51)	24.5 [19.7–35.8] (17.52–392)
**CRP**	mg/l	0.5 [0.3–0.6] (0–0.82)	2.2 [1.4–3.4] (0.83–5.99)	11.5 [7.8–21.2] (6–447.56)
**TnT**	ng/l	6.3 [5.3–6.9] (3–7.48)	11.3 [9.2–14.1] (7.50–18.59)	25.1 [21.5–34.1] (18.62–1084)
**D-dimer**	ng/ml	0.04 [0.02–0.05] (0–0.07)	0.17 [0.11–0.25] (0.08–0.41)	0.75 [0.54–1.23] (0.42–6.34)
**ESM1**	ng/ml	1.4 [1.2–1.5] (0.84–1.53)	2.1 [1.8–2.3] (1.54–2.85)	3.8 [3.2–6.0] (2.85–24.62)
**FABP3**	ng/ml	22.5 [20.1–24.1] (11.44–25.06)	32.1 [28.7–36.1] (25.06–42.5)	51.4 [46.6–60.8] (42.55–335.66)
**FGF23**	pg/ml	95.0 [85.8–102.4] (41.02–108.47)	154.8 [131.4–187.5] (108.48–237.96)	334.3 [275.9–474.6] (238.98–3901.60)
**GDF15**	pg/ml	776.3 [664.3–848.2] (400–912)	1355.5 [1122–1687.75] (913–2194)	2994 [2511–3980] (2199–18255)
**IGFBP7**	ng/ml	82.1 [76.7–85.2] (42.90–87.95)	102.4 [95.8–109.5] (88.03–121.93)	138.5 [129.6–159.3] (121.98–355.46)
**IL-6**	pg/ml	1.5 [1.5–1.5] (1.5–1.51)	2.63 [2.03–3.42] (1.52–4.72)	7.44 [5.67–11.03] (4.74–1376)
**NT-proBNP**	pg/ml	89 [61–123] (5.51–146.6)	459. [264–744] (147.2–1179)	1838 [1431–2482] (1183–25174)
**sCr**	μmol/l	59 [54–63] (36–65)	79 [72–86] (66–96)	109 [100–120] (97–577)

Values given as concentrations (before transformation and outlier management) as median and interquartile range (IQR) as a range from 25th to 75th percentile and range. ANGPT2, angiopoietin 2; BMP10, bone morphogenetic protein 10; CA125, cancer antigen 125; CRP, C-reactive protein; ESM1, endothelial specific molecule 1; FABP3, fatty acid binding protein 3; FGF23, fibroblast growth factor 23; GDF15, growth differentiation factor 15; IGFBP7, insulin-like growth factor binding protein 7; IL-6, interleukin-6; NT-proBNP, *n*-terminal pro-B-type natriuretic peptide; sCr, serum creatinine; TnT, cardiac troponin. Values of 0 indicate actual measurements below lower limit of quantification.

**Table 2:** Clinical characteristics stratified by discretized BMP10 in three groups according to quintiles

	Discretized BMP10 by percentile			
Variable	Low 1st quintile	Intermediate 2nd – 4th quintile	High 5th quintile	*P*
**BMP10 in** ng/ml **(median [IQR]; (range))**	1.62 [1.51–1.71]; (1.05–1.77)	2.11 [1.96–2.29]; (1.77–2.54)	2.89 [2.68–3.18]; (2.54–4.96)	
n **patients**	318	953	315	
**Treatment type aka Random group (%)**				0.167
**Usual care**	147 (46)	477 (50)	162 (51)	—
**Early treatment**	171 (54)	476 (50)	153 (49)	—
**Female sex (%)**	86 (27)	445 (47)	182 (58)	< 0.001
**Age [median (IQR)]**	69 [62, 74]	71 [66, 75]	73 [68, 78]	< 0.001
**BMI (median [IQR])**	29.7 [26.6, 32.8]	28.7 [25.6, 32.3]	27.8 [24.5, 31.3]	< 0.001
**Sinus rhythm at baseline (%)**	236 (74)	542 (57)	112 (36)	< 0.001
**Sinus rhythm at FU 12 (%)**	243 (76)	664 (70)	174 (55)	< 0.001
**Sinus rhythm at FU24 (%)**	234 (74)	597 (63)	157 (50)	< 0.001
**Diastolic BP [mean (SD)]**	80.73 (11.41)	81.59 (12.30)	81.30 (11.64)	0.331
**Systolic BP [median (IQR)]**	135 [125, 145]	136 [124, 150]	135 [120, 145]	0.753
**CHA_2_DS_2_-Vasc-score** **[mean (SD), median (IQR)]**	3 (1), 3 [2, 4]	3 (1), 3 [2, 4]	4 (1), 4 [3, 5]	< 0.001
**CKD (%)**	26 (8)	114 (12)	55 (18)	0.010
**AF type**				
**First episode**	120 (38%)	326 (34%)	114 (36%)	Reference
**Paroxysmal**	145 (46%)	365 (38%)	80 (25%)	0.495
**Persistent**	53 (17%)	262 (28%)	121 (38%)	< 0.001
**Previous stroke or TIA (%)**	44 (14)	116 (12)	35 (11%)	0.249
**Diabetes (%)**	90 (28)	223 (23)	83 (26)	0.088
**COPD (%)**	20 (6)	72 (8)	32 (10)	0.476
**NYHA at baseline (%)**				
**I**	22 (7)	103 (11)	45 (14)	Reference
**II**	67 (21)	179 (19)	78 (25)	0.146
**III**	18 (6)	24 (3)	18 (6)	0.026
**No heart failure**	211 (66)	647 (68)	174 (55)	0.030
**EHRA at baseline (%)**				
**I**	94 (30)	290 (30)	84 (27)	Reference
**II**	144 (45)	468 (49)	148 (47)	0.692
**III**	51 (16)	135 (14)	58 (18)	0.805
**IV**	5 (2)	9 (1)	3 (1)	0.301
**Missing**	24 (8)	51 (5)	22 (7)	
**Left atrial diameter (LAD) mm [median (IQR)]**	43 [39, 48]	42 [38, 47]	42 [38, 47]	0.360
**GFR (CKD-EPI) in mL/min/1.73 m^2^ [median (IQR)]**	79 [67, 90]	76 [64, 87]	70 [57, 81]	< 0.001

Dichotomous and categorical (nominal or ordinal) scaled variables are reported as absolute numbers and percentages in parentheses. Continuous scaled variables are reported as mean and standard deviation (SD) if data are approximately normally distributed and as median and interquartile range (IQR) if data are non-normally distributed. AF, atrial fibrillation; BMI, body mass index; BMP10, bone morphogenetic protein 10; BP, blood pressure; CKD, chronic kidney disease; COPD, chronic obstructive lung disease; EPI, Epidemiology Collaboration; EHRA, European Heart Rhythm Association; EQ5D, EuroQol-5− Dimension; GFR (CKD-EPI), glomerular filtration rate (Chronic Kidney Disease Epidemiology Collaboration equation); IQR, interquartile range; NYHA, New York Heart Association. *P*-values were derived from mixed logistic regression models per variable with study centre as random effect, first quintile (vs. second to fifth quintile) as dependent variable and the trait as independent variable. For categorical multi-class variables, the first class served as reference for the model.

**Table 3:** BMP10 concentrations and number of events in each BMP10 discretized group. Numbers indicate patients with events (percentage))

	BMP10 low		BMP10 intermediate		BMP10 high	
	EAST-AFNET 4 ERC	EAST-AFNET 4 usual care	EAST-AFNET 4 ERC	EAST-AFNET 4 usual care	EAST-AFNET 4 ERC	EAST-AFNET 4 usual care
n **(patients)**	171	147	476	477	153	162
**BMP10 in** ng/ml **(median [IQR])**	1.61 [1.52, 1.69]	1.62 [1.50, 1.71]	2.12 [1.96, 2.28]	2.10 [1.94, 2.28]	2.92 [2.69, 3.24]	2.87 [2.68, 3.11]
Components of the primary outcome						
**Cardiovascular death**	7 (4%)	4 (3%)	18 (4%)	32 (7%)	10 (7%)	19 (12%)
**Stroke**	4 (2%)	1 (1%)	12 (3%)	20 (4%)	2 (1%)	12 (7%)
**Unplanned hospitalization for heart failure**	10 (6%)	7 (5%)	37 (8%)	57 (12%)	25 (16%)	29 (17%)
**Unplanned hospitalization for acute coronary syndrome**	8 (5%)	4 (3%)	13 (3%)	28 (6%)	3 (2%)	7 (4%)
Components of the safety outcome						
**Death**	13 (8%)	9 (5%)	35 (7%)	48 (10%)	19 (12%)	21 (13%)
**Severe adverse events related to rhythm control**	6 (4%)	0 (0%)	26 (5%)	9 (2%)	5 (3%)	3 (2%)
**Major bleeding**	0 (0%)	0 (0%)	0 (0%)	0 (0%)	1 (1%)	0 (0%)
Secondary outcome						
**Sinus rhythm at BL**	122 (71%)	114 (78%)	272 (57%)	270 (57%)	58 (38%)	54 (33%)
**Sinus rhythm at 12 months** **Change to BL**	138 (81%) +10%	105 (71%) −7%	379 80%) +33%	285 (60%) +3%	108 (71%) +33%	66 (41%) +8%
**Sinus rhythm at 24 months**	136 (80%) +9%	98 (67%) −11%	356 (75%) +18%	241 (51%) −6%	94 (61%) +23%	63 (40%) +7%
**Change to BL**						

BL, baseline; ERC, early rhythm control; SAE, serious adverse event of special interest related to rhythm-control therapy.

### Sensitivity analyses

**Figure 5:**
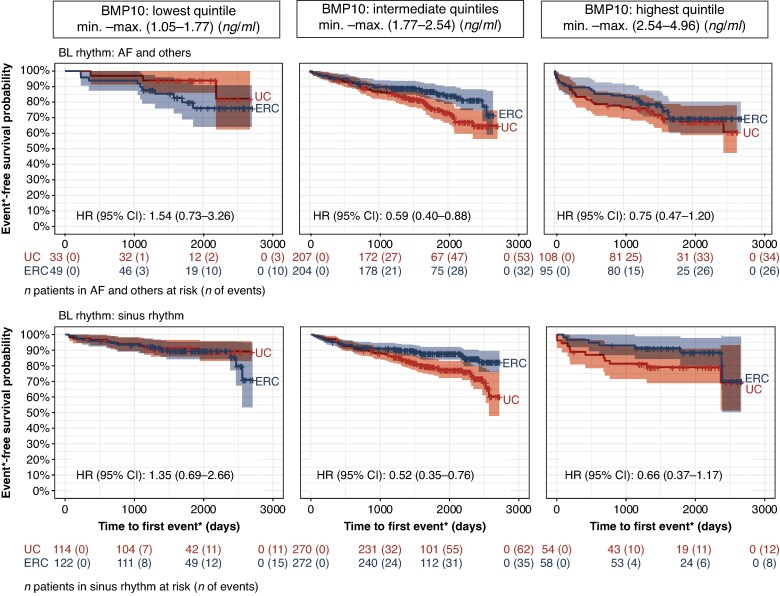
Two-way interaction analysis between discretized BMP10 and heart rhythm at baseline with the treatment group. AF, atrial fibrillation; BL, baseline; ERC, early rhythm control; HR, hazard ratio; UC, usual care.

When analysed as continuously scaled variables, none of the biomolecule concentrations analysed interacted with ERC (see Supplementary material online, *Table S2*). BMP10 is associated with AF^20^ and patients in the low BMP10 group were more often enrolled in sinus rhythm (*Table 2*). Therefore, an additional model integrating rhythm at baseline as a second interaction term was constructed. The event-free survival probability plots stratified for discretized BMP10 show a similar pattern of interaction signal with treatment groups for patients in sinus rhythm and patients in AF during blood draw (*Figure 5*). BMP10 concentrations are higher in women than in men.^19^ When including sex as another interaction term, patients in the lowest BMP10 quintile keep numerically lower event rates with UC (see Supplementary material online, *Figure S3*, Supplementary material online, *Table S3*). As a sensitivity analysis, we further combined intermediate and high quintile groups to dichotomize only two distinct patient clusters, one holding patients with BMP10 concentrations in the first quintile (low) and one with BMP10 concentrations ranging from the second to fifth quintile (intermediate to high). The signal was similar (*P* for interaction = 0.012, *q* for interaction = 0.169), with treatment effect of ERC in the low BMP10 concentration group: HR (95% CI) 1.40 (0.73–2.71), *P*: 0.293 and in the group with intermediate to high BMP10 concentrations HR (95% CI) 0.60 (0.46–0.76), *P*: <0.001 (see Supplementary material online, *Figure S4*).

### Replication

**Figure 6:**
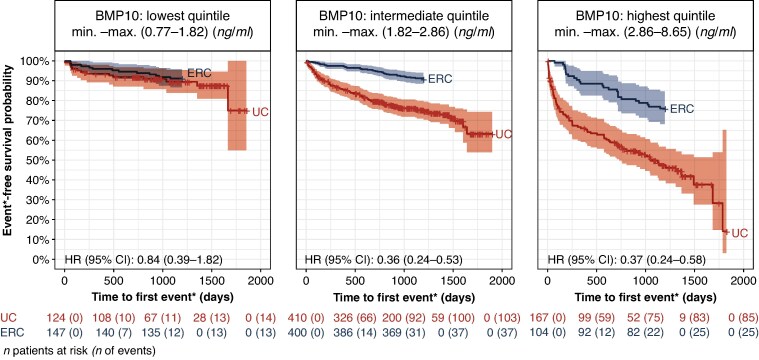
Sensitivity analyses and external replication. AF, atrial fibrillation; BL, baseline, ERC, early rhythm control; HR, hazard ratio; UC, usual care. Effect of BMP10 (low, intermediate, and high BMP10 concentrations) on outcomes with early rhythm control in the derived validation dataset created by combining the BBC-AF (UC) data set and TRUST snapshot (ERC). Estimated event-free survival probability for the *first primary outcome stratified by discretized BMP10 over the follow-up time. Hazard ratios stem from Cox proportional hazard models.

Exploratory replication in independent observational cohorts, one predominantly treated with rate control (usual care, BBC-AF) and the other predominantly treated with AF ablation (ERC, TRUST) replicated the signal of BMP10 concentrations on the effect estimates of ERC therapy. Patients in the lowest BMP10 quintile group have a modelled HR of 0.86 (0.40–1.86, *P* = 0.704) in the combined BBC-AF and TRUST data with outcome differences between usual care and ERC. Patients with intermediate or high BMP10 concentrations show separation of the Aalen-Johansen curves with fewer events with rhythm control. Participants in the intermediate quintile group have a modelled HR of 0.35 (0.24–0.52), and patients in the highest BMP10 group a HR of 0.37 (0.24–0.58, both *P* < 0.001, *Figure 6*, Supplementary material online, *Figure S5*). FGF23 and IGFBP7, in contrast, showed largely overlapping treatment effects, replicating the lack of interaction signal in EAST-AFNET 4 (see Supplementary material online, *Figure S5*).

### Safety outcome

As there was no overall difference in safety outcome between randomized groups—a composite of death from any cause, stroke, or prespecified SAE capturing complications of rhythm-control therapy, the safety outcome was analysed as a plausibility check. Two of the analysed biomolecules showed an interaction signal. Those are endothelial cell-specific molecule-1 (ESM1) and IGFBP7. ESM1 showed heterogeneity with *P* for interaction = 0.03, *q* for interaction = 0.25 and IGFBP7 with *P* for interaction = 0.04, *q* for interaction = 0.25. For ESM1: For participants in the first (low) quintile HR (95% CI): 1.49 (1.00–3.75), *P* = 0.049, for participants in the second–fourth quintile HR (95% CI): 0.73 (0.52–1.03), *P*: 0.071 and for participants in the fifth quintile HR (95% CI): 0.88 (0.54–1.45), *P* = 0.627. For patients with low IGFBP7 concentrations estimated treatment effect is HR (95% CI): 1.62 (0.78–3.38), for patients with medium concentrations HR (95% CI): 0.97 (0.68–1.38), and for participants with highest IGFBP7 values HR (95% CI): 0.60 (0.39–0.92). In patients in the lowest BMP10 quintile, there are numerically fewer safety outcomes in patients randomized to UC (see Supplementary material online, *Figure S2*, *P* for interaction = 0.07, *q* for interaction = 0.25).

## Discussion

This exploratory analysis of preselected mechanistic biomolecules in the EAST-AFNET 4 biomolecule study supports the effectiveness and safety of ERC in patients with high or low concentrations of biomolecules reflecting cardiac strain and damage, inflammation, ageing, and fibrosis. This supports the emerging paradigm shift towards the use of ERC therapy to reduce cardiovascular events and ongoing clinical trials evaluating ERC or early AF ablation for outcome reduction in high-risk populations, including EASThigh-AFNET 11 (NCT06324188), CABA-HFPEF DZHK27 (NCT05508256),^31^ EAST STROKE (NCT05293080), and others.^32^ The findings also confirm a recent analysis suggesting effectiveness and safety of ERC with and without atrial cardiomyopathy.^12^ Within the limitations of an exploratory analysis with exploratory replication in a combined data set, the effectiveness of ERC therapy may be reduced in patients with very low BMP10 concentrations. The nominal significance and the exploratory nature of all analyses highlight their hypothesis-finding nature. This warrants further research.

BMP10 is an atrial-specific, secreted protein.^33^ Elevated concentrations of BMP10 in peripheral blood are associated with AF^20,33–35^ and with AF-related complications such as stroke^36,37^ and heart failure.^37,38^ High atrial rates cause BMP10 release from engineered atrial tissue, which then promotes gene expression linked to AF and heart failure.^39^ Previous work already showed that elevated concentrations of BMP10 are associated with recurrent AF.^33^ Prior analyses demonstrated that lower BMP10 concentrations are associated with a higher likelihood of maintaining sinus rhythm.^16^ The present analysis suggests that these patients may derive less clinical benefit from ERC. One possible explanation is that patients with low BMP10 represent a subgroup with a higher intrinsic probability of maintaining sinus rhythm, thereby reducing the incremental benefit of an ERC strategy. Conversely, patients with higher BMP10 levels may have more advanced atrial disease and a lower likelihood of spontaneous rhythm control, but greater potential to benefit from active rhythm control in terms of clinical outcomes. This is potentially reflected in the number of patients in sinus rhythm in the BMP10 and treatment groups (*Table 3*, replication cohorts *Table 4*). This information indeed suggests that the potentially weaker effectiveness of ERC in patients with very low BMP10 concentrations could be due to a lower AF burden.^32,40^ The lack of interaction for NT-proBNP in the present analysis highlights that biomarkers associated with rhythm outcomes do not necessarily overlap with treatment-modifying biomarkers. Recent experimental results linking BMP10 secretion to high atrial rates and demonstrating an effect on ventricular contractility^39^ and our present results support a possible role for BMP10 in arrhythmia-induced cardiomyopathy in patients with AF.^41^ BMP10 concentrations were higher in women in this study and in a recent report.^19^ Correcting for rhythm and sex did not diminish the potential interaction signal between BMP10 and ERC. The exploratory replication provides a first confirmation that patients with low BMP10 concentrations are at low risk of cardiovascular events, consistent with an analysis of stroke rates in the direct oral anticoagulant registration trials.^36^ These findings call for more research into the function of BMP10 to understand possible links between BMP10 and rhythm control therapy.^42^ In the replication data set, similar signals were found with BMP10 and with very low concentrations of ANGPT2 and NT-proBNP (see Supplementary material online, *Figure S5*). The same biomolecules predict sinus rhythm after AF ablation in the AXAFA-AFNET 5^43^ and EAST-AFNET 4^16^ trials and in a Swiss cohort.^34^ More research is needed to understand the potential mechanisms underlying these collinearities that may be partially due to underlying biomedical mechanisms,^14^ atrial cardiomyopathy,^12^ and to AF burden.^40^ Such understanding may provide a foundation for precision medicine to select rhythm control therapy in patients with AF.^44,45^

**Table 4:** Number of events in each BMP10 discretized group in the two replication cohorts

	BMP10 low		BMP10 intermediate		BMP10 high	
	TRUST snapshot (ERC)	BBC-AF (usual care)	TRUST snapshot (ERC)	BBC-AF (usual care)	TRUST snapshot (ERC)	BBC-AF (usual care)
**Cardiovascular death**	7 (4%)	4 (3%)	18 (4%)	32 (7%)	10 (7%)	19 (12%)
**Stroke**	4 (2%)	1 (1%)	12 (3%)	20 (4%)	2 (1%)	12 (7%)
**Unplanned hospitalization for heart failure**	10 (6%)	7 (5%)	37 (8%)	57 (12%)	25 (16%)	29 (18%)
**Unplanned hospitalization for acute coronary syndrome**	8 (5%)	4 (3%)	13 (3%)	28 (6%)	3 (2%)	7 (4%)
**Death**	13 (8%)	9 (6%)	38 (8%)	58 (12%)	19 (12%)	26 (16%)
**Major bleeding**	0 (0%)	0 (0%)	0 (0%)	0 (0%)	1 (1%)	0 (0%)

BBC-AF (resembling usual care) and TRUST snapshot (resembling early rhythm control, mainly AF ablation). Numbers indicate patients with events (percentage).

### Strengths and limitations

Strengths of the analysis are the locked data set of a randomized controlled trial with adjudicated long-term cardiovascular outcomes, the central quantification of biomolecules with high precision blind to this analysis and the pre-specified analysis plans. The study has several limitations. First, this is an exploratory analysis in a relatively small number of patients. The reversal of the Aalen–Johansen curves in patients with a low BMP10 concentration is visually striking but could be play of chance. Validation of our observation and further research into the mechanisms and features that lead to low BMP10 concentrations are needed to further explore a possible association of BMP10 and rhythm control therapy success rate. Second, the split of patients into groups by biomolecule by quintiles is arbitrary, although this is common practice in analyses of risk based on quantitative parameters, e.g. in common gene variant analyses.^7^ Third, there were insufficient number of outcome events for an internal discovery-validation split dataset in the EAST-AFNET 4 biomolecule study. Fourth, rhythm control therapy selection in EAST-AFNET 4 reflects the treatment patterns at the time of the trial (2011–2020), mainly delivered using sodium channel blockers^4^ and potassium channel blockers.^46^ Changes in the use of antiarrhythmic drugs and a growing role for AF ablation may affect the findings in contemporary cohorts, including the TRUST snapshot used for replication in this analysis. Fifth, anticoagulation therapy lowers D-dimer concentrations.^47^ A high proportion of participants in the EAST-AFNET 4 trial were started on anticoagulation using vitamin K antagonists or DOACs after inclusion into the study (92%),^1^ but not necessarily prior to the baseline blood sample. Therefore, analysis of D-dimer concentrations has to be treated with extra caution. Sixth, there is no other trial that contains information on BMP10, has a randomized rhythm control therapy intervention, and contains information on outcomes. We therefore replicated the findings in a constructed data set containing data from two cohorts (BBC-AF and TRUST). The indirect comparison of two observational data sets, one treated according to usual care, and one treated with ERC, carries additional confounding risks. Seventh, the absence of interaction when modelling BMP10 as a continuous variable, contrasted with an interaction signal in categorical analyses, suggests potential cut-point dependence or alternatively a difference observed by chance. Categorization of continuous biomarkers may introduce arbitrary thresholds and contribute to inflation of type I error. Therefore, these findings should be interpreted with caution and considered hypothesis-generating. Eighth, current data are insufficient to infer mechanism and observational biomarker associations do not support pathway-level conclusions; therefore, more research is needed.

### Conclusion

Our hypothesis-generating results confirm the effectiveness of ERC therapy in patients with high and low blood plasma concentrations of thirteen biomolecules reflecting different disease mechanisms, including cardiac strain and damage, inflammation, ageing, and fibrosis. A single exploratory signal (BMP10) suggests an attenuated effect of ERC in patients with low BMP10 concentrations. Further research into the function of BMP10 is needed.

## Supplementary Material

euag149_Supplementary_Data

## Data Availability

Data will be made available upon reasonable request. Parts of the code will be made available at https://github.com/UCCSHH.
