# Supplementary material for: Interactions of blood biomolecules with early rhythm control in atrial fibrillation patients: exploratory analysis of the EAST-AFNET 4 biomolecule study
Source: Europace. 2026 Jun 23;28(7):euag149. doi: 10.1093/europace/euag149 (PMC13390925; doi:10.1093/europace/euag149)
Supplement: euag149_Supplementary_Data [file euag149_supplementary_data.zip › ATT00002.htm]

  

---

Universitätsklinikum Hamburg-Eppendorf; Körperschaft des öffentlichen Rechts; Gerichtsstand: Hamburg | www.uke.de  
Vorstandsmitglieder:
Prof. Dr. Christian Gerloff (Vorsitzender), Joachim Prölß, Prof. Dr.
Blanche Schwappach-Pignataro, Corinna Wriedt

---
