# Supplementary material for: Interactions of blood biomolecules with early rhythm control in atrial fibrillation patients: exploratory analysis of the EAST-AFNET 4 biomolecule study
Source: Europace. 2026 Jun 23;28(7):euag149. doi: 10.1093/europace/euag149 (PMC13390925; doi:10.1093/europace/euag149)
Supplement: euag149_Supplementary_Data [file euag149_supplementary_data.zip › east-afnet-biomarker-ss-sap-v05.pdf]

Statistical Analysis Plan (SAP)

for

Early rhythm control therapy in patients with atrial fibrillation

EAST-AFNET 4

Circulating biomolecule sub-study

2. Synopsis of study design and procedures

Study Design

3. Description of Analyses

Deviations from protocol

As participation in the circulating biomolecules sub-study is not randomized, but open to all participants independent of their intervention, deviations from the protocol do not apply. Regarding the primary analysis, deviations from the study protocol are collected in a standardized manner.

Analysis Population

The EAST – AFNET 4 study offered all participants participation in a biosample sub-study. Due to regulatory delays, this offer could not be made to the first 399 patients. The analysis population will consist of all patients randomized in the EAST – AFNET 4 trial in whom biosamples were collected.
For comparison of the effect of the randomized intervention, early rhythm control, we will compare intention to treat populations as a primary analysis.
The analysis population will include those patients from the original study who agreed to also contribute to the circulating biomolecules sub-study. These are 1586 patients of which approximately 50% are women and also around 50% received usual care against about 50% that received early treatment.
We will compare clinical characteristics of the sub-study population with the patients not participating in the sub-study to identify potential differences.

Aim of the study

The study aims to identify circulating biomolecules associated with the **severity level of heart disease**. Promising candidate circulating biomolecules were identified using a modified Delphi approach. Details have been published, [7], [8], [9]. For this purpose, different multivariate analyses are performed. In addition, different machine learning (ML) models will be used to predict the **dependent variables**:

- **1st co-primary outcome** – which is a composite of the variables:
  - cardiovascular death
  - stroke / transient ischemic attack
  - hospitalization
    - due to worsening of heart failure or
    - due to acute coronary syndrome

  is a time-to-event endpoint consisting of:

  - a categorical, nominal scaled variable that can take the two expressions true or false

    and

  - the time from admission of the participant in the study till observation of the event.

- **2nd co-primary outcome** – 'nights spend in hospital per year'

  is a numeric, discrete scaled variable that ranges from *0* to *n* number of nights. We will add one night to each value to enable log transformation in addition to using the collected number of nights spent in hospital.

- **Primary safety outcome** – which is a composite of death, stroke, and serious adverse events of rhythm control therapy.

To increase power and to explore the effect on all events, recurrent events will be analyzed in a separate analysis. In addition, circulating biomolecules predictive of components of the primary outcome will be analyzed. We will also determine biomolecule concentrations associated with the primary safety outcome, its components, and biomolecule concentrations associated with major bleeding (captured as SAE). To determine factors that affect the primary outcome, we plan an extended multivariate analysis including clinical parameters associated with the primary outcome (see Kirchhof et al NEJM 2020, supplementary data and Metzner et al Europace 2021) and biomolecule concentrations.

- **Secondary outcomes of interest** – Relevant secondary outcomes were estimated at the two-year follow-up visit. These include cognitive function, estimated by the MoCA score; left ventricular function, estimated as ejection fraction in percent; quality of life estimated by the EQ-5D visual analog scale (0-100) and by the SF-12 questionnaire; and rhythm (categorical: sinus rhythm, atrial fibrillation, other). Models will be built to predict these outcomes. Recurrent AF will be defined in two ways: In all patients, AF at the two-year ECG will be used to define recurrent AF. In patients receiving early rhythm control therapy, an unscheduled visit due to AF will be used to define recurrent AF. At a later stage, the analyses of the telemetric ECGs planned in Maastricht will be used to define recurrent AF in patients randomized to early rhythm control. The latter two definitions create an imbalance between the two randomized groups. For continuous parameters, models will be applied to three values, namely 1. The baseline value, 2. The value at two years, and 3. The change between baseline and two years.

- **Specific hypotheses**. Based on our biomedical knowledge, we developed specific hypotheses that will be tested in the EAST – AFNET 4 biomarker sub-study. These include the following:
    1. BMP10 concentrations are associated with recurrent AF and affect recurrent AF differentially depending on the use of sodium channel blockers (flecainide, propafenone).
    2. FGF23 concentrations are associated with recurrent AF.
    3. A combination of ANG2, FGF23, and BMP10 predicts recurrent AF better than natriuretic peptides.
    4. ESM1 concentrations are associated with stroke (both prior stroke at baseline and future stroke during FU) and with the composite of stroke and cardiovascular death
    5. The ABC bleeding score is associated with major bleeding
    6. The ABC stroke score is associated with stroke and with the composite of stroke and cardiovascular death
    7. BNP is associated with heart failure hospitalizations and with the composite of death or heart failure hospitalization
    8. Angiopioetin 2, FGF23 (alone or in combination or in combination with ESM1) are strong predictors for stroke or stroke and ACS, or stroke + ACS + CV death

The **independent** variables are:

- The 14 circulating biomolecules:
  - ANG2
  - BMP10
  - Cancer-Antigen 125 (CA-125)
  - Cardiac C-Reactive Protein High Sensitive
  - Creatinine (enzymatic determination)
  - D-Dimer [Explicit units would be "ug FEU/mL]
  - ESM1
  - FBP3
  - FGF23
  - Growth Differentiation Factor-15
  - Interleukin-6
  - IGFBP7
  - NT-proBNP
  - Troponin T high sensitive (cTnT-hs)

  all numeric, continuous scaled

To enable evaluation of these circulating biomolecules in context with clinical information, a list of parameters that are associated with the primary outcome will be included in the analysis. A preliminary list will be taken from the existing analyses in the trial. If there are too many candidates, we will do some variable reduction to discard “redundant” variables without using the association with the endpoints to inform that decision (e. g. correlations, PCA, etc.).

See supplement to the main results publication for a list of preselected parameters.

- additional **clinical** parameters collected at baseline:
  - age

    numeric, discrete scaled
  - gender

    categorical, nominal scaled – dichotomous
  - random group

    categorical, nominal scaled – dichotomous
  - BMI

    numeric, continious scaled
  - Heart Failure, determined using heart failure symptoms and LVEF

    categorical, nominal scaled – dichotomous

- EHRA Score at baseline
  Categorical, ordinal scaled
- Center Type (D-Site or A-Site)
  categorical, nominal scaled – dichotomous
- Diabetes mellitus
  categorical, nominal scaled – dichotomous
- History of CAD (MI, CABG or PCI)
  categorical, nominal scaled – dichotomous
- Prior stroke or TIA
  categorical, nominal scaled – dichotomous
- AF pattern
  categorical, nominal scaled
- MoCA Score at baseline
  numeric, discrete scaled
- EQ5D
  numeric, discrete scaled
- 'Systolic blood pressure [mmHg]'
  numeric, discrete scaled
- 'Diastolic blood pressure [mmHg]'
  numeric, discrete scaled
- 'Diastolic LA diameter (maximal diameter) [mm]'
  numeric, discrete scaled
- 'Systolic LA diameter (minimum diameter), optional [mm]'
  numeric, discrete scaled
- Chronic kidney disease (MDRD stage)
  categorical, ordinal scaled
- Heart failure (NYHA classification)
  categorical, ordinal scaled

Types of Analysis

Descriptive Analytics

For all numeric variables the following univariate statistics will be provided:

- mean
- standard deviation
- median
- 25th and 75th quartiles

For all categorical variables the following statistics will be provided:

- absolute frequencies
- relative frequencies

Inference Analytics

It is expected that clinical parameters and biomolecule concentrations will interact in complex ways. These multiple interactions are not well captured by classical regression analyses. On the other hand, multiple regression remains the ´gold standard´ in the field. To reflect these complexities and demands, we will train four types of models separately for the 1st primary outcome, the 2nd primary outcome, and the primary safety outcome. Those model types include statistical learning (SL), machine learning, and deep learning (DL) approaches. Furthermore, each of the 3 * 4 = 12 models will be trained with clinical confounders and with clinical confounders combined with circulating biomolecules. This gives us the ability to eventually compare 24 models and their reflection of interactions between the clinical parameters and biomolecule concentrations.

- A time-to-event machine learning problem to be solved by different candidate models to be compared against each other. These are namely:
- For 1st primary outcome, 1st primary safety outcome and their components
  - Cox regression – SL - classic approach
  - random survival forest – ML – Tree-based ensemble (bagging)
  - gradient boosted model – ML – Tree-based ensemble (boosting)
  - artificial neural network – DL – multi layer perceptron
- A regression ML-problem to be solved by the following candidate models:
- For 2nd co-primary outcome
  - multiple linear regression – SL - classic approach
  - random forest – ML – Tree-based ensemble (bagging)
  - gradient boosted model – ML – Tree-based ensemble (boosting)
  - artificial neural network – DL – multi layer perceptron

Model selection

Between model selection

The best performing model for each of the three ML-problems will be used for further analysis of risk factors. To identify the best model for the regression ML-problem we are going to use **Akaike Information Criteria - AIC** as it is applicable to linear models and non-linear models as well and takes into account model performance and its complexity.

For identifying the best performing time-to-event ML-model we will use the **c-index** and the **Brier Score**.

The information criteria scores will be obtained from applying the model to a hold-out/test dataset of the complete dataset.

Inside model selection

To find the optimal hyper-parameters (not features/variables to use) for each model (except multiple linear - / Cox regression as they do not have hyper-parameters) **Random Grid Search Cross Validation** will be used. As this technique is able to identify near optimal parameters in reasonable time.

Identification of relevant circulating biomolecules as risk factors

To identify the circulating biomolecules that can be used as robust explanatory variables i. e. risk factors **permutation feature importance [1]** will be used as it is a model agnostic technique.

Data preprocessing

Categorical variables

The categorical variables are all dichotomous therefore no grouping is applied. All categorical variables will be one-hot-encoded i.e. **dummy variables** will be created.

Numerical variables

Numerical variables will be **normalized** before being fed into the models for training and testing. Also log-transformation of numeric variables will be applied. As these transformations are crucial for the success of Algorithms that rely on stochastic gradient decent like artificial neural networks, they are not necessary preprocessing steps for tree-based models.

We are going to apply these transformations anyway to compare their impact on the prediction power.

All variables

A 20 % **hold-out dataset** will be used for evaluating the final model that only relies on the most relevant variables and has been trained on the remaining 80 % of the dataset. At model training time the training set will be further split into **k-fold-cross validation** datasets. The split will be **stratified** for the dependent variable.

Handling errors and missing data

For one proband there is a special entry for Variable 'ANG2'. The entry value is 'BLQ' – that stands for Below Limit of Quantitation. Normally those entries will be dealed with as proposed by Bergstrand et al [2]. As we are facing only one entry of this type we are going to set this value to the smallest 'ANG2' value available in the whole dataset.

The remaining dataset has a sample-size of 1585 and missing entries as follows:

- Diastolic LA diameter 119
- EHRA score at baseline 97
- MOCA Score 42
- LVEF 5
- BMI 4

The missing entries will be imputed by SciKit-learn's Iterative Imputer. [3] [4].

Handling unbalanced datasets

We want balanced datasets which means the relation of female to male patients inside the two treatment groups should be equal. Also the number of patients that saw a worsening in heart failure and the ones that did not needs to be balanced. For this purpose, we are going to use Synthetic Minority Oversampling technique – **SMOTE [5]** which uses unsupervised machine learning for clustering to create new augmented samples similar to those of the minority group.

Documentation and Software

Data preparation and analysis will be performed using

- **python Version 3.8.1**

and the following **packages** and their **dependencies**:

  - **pandas version 1.2.4**
  - **scikit-learn version 0.24.2**
  - **scikit-survival version 0.15.1**
  - **TensorFlow version 2.6.0**
  - **PyTorch version 1.10**
  - **pycox version 0.2.2**

- **R Version 4.1.1**

Further analysis

The whole dataset will be further split into **women and men** and independent models could be trained and tested for each sub-dataset to further examine the different impact of various circulating biomolecules for men and women.

The dataset could also be split into different **age-groups** to better understand the interaction between circulating biomolecules, age and the severity level of heart disease.

There will be additional groups, by kidney function class, symptom class, heart failure (yes/no), comorbidity index (chadsvasc 4 or more compared to chadsvasc 3 or less)

As we are solving two distinct tasks (prediction of the nights spend in hospital and the time to event analysis) that do not only rely on the same dataset but are also likely to be correlated with each other a **Multi-task learning** approach could be interesting. Building ANNs with multiple outputs for classification and regression at the same time are well known. However, combing time-to-event analysis with a regression or classification ML-task seems to be of ongoing research.

References

[1] Altmann, A., Toloşi, L., Sander, O., Lengauer, T., 2010. Permutation importance: a corrected feature importance measure. Bioinformatics 26, 1340–1347. https://doi.org/10.1093/bioinformatics/btq134

[2] Bergstrand, M., Karlsson, M.O., 2009. Handling data below the limit of quantification in mixed effect models. AAPS J 11, 371–380. https://doi.org/10.1208/s12248-009-9112-5

[3] Buck, S.F., 1960. A Method of Estimation of Missing Values in Multivariate Data Suitable for use with an Electronic Computer. Journal of the Royal Statistical Society. Series B (Methodological) 22, 302–306.

[4] Buuren, S. van, Groothuis-Oudshoorn, K., 2011. mice: Multivariate Imputation by Chained Equations in R. Journal of Statistical Software 45, 1–67. https://doi.org/10.18637/jss.v045.i03

[5] Chawla, N.V., Bowyer, K.W., Hall, L.O., Kegelmeyer, W.P., 2011. SMOTE: Synthetic Minority Over-sampling Technique. arXiv:1106.1813 [cs]. https://doi.org/10.1613/jair.953

[6] Chua, W., Law, J.P., Cardoso, V.R., Purmah, Y., Neculau, G., Jawad-Ul-Qamar, M., Russell, K., Turner, A., Tull, S.P., Nehaj, F., Brady, P., Kastner, P., Ziegler, A., Gkoutos, G.V., Pavlovic, D., Ferro, C.J., Kirchhof, P., Fabritz, L., 2021. Quantification of fibroblast growth factor 23 and N-terminal pro-B-type natriuretic peptide to identify patients with atrial fibrillation using a high-throughput platform: A validation study. PLOS Medicine 18, e1003405. https://doi.org/10.1371/journal.pmed.1003405

[7] Fabritz, L., Guasch, E., Antoniades, C., Bardinet, I., Benninger, G., Betts, T.R., Brand, E., Breithardt, G., Bucklar-Suchankova, G., Camm, A.J., Cartlidge, D., Casadei, B., Chua, W.W.L., Crijns, H.J.G.M., Deeks, J., Hatem, S., Hidden-Lucet, F., Kääb, S., Maniadakis, N., Martin, S., Mont, L., Reinecke, H., Sinner, M.F., Schotten, U., Southwood, T., Stoll, M., Vardas, P., Wakili, R., West, A., Ziegler, A., Kirchhof, P., 2016. Expert consensus document: Defining the major health modifiers causing atrial fibrillation: a roadmap to underpin personalized prevention and treatment. Nat Rev Cardiol 13, 230–237. https://doi.org/10.1038/nrcardio.2015.194

[8] Prebianca, F., Marcondes, D.W.C., Albuquerque, H.A., Beims, M.W., 2019. Exploring an experimental analog Chua's circuit. Eur. Phys. J. B 92, 134. https://doi.org/10.1140/epjb/e2019-100097-4
