# Supplementary material for: Interactions of blood biomolecules with early rhythm control in atrial fibrillation patients: exploratory analysis of the EAST-AFNET 4 biomolecule study
Source: Europace. 2026 Jun 23;28(7):euag149. doi: 10.1093/europace/euag149 (PMC13390925; doi:10.1093/europace/euag149)
Supplement: euag149_Supplementary_Data [file euag149_supplementary_data.zip › Suppl tables&figuresEUPC-D-26-00422R1.pdf]

Supplemental Material

Interactions of blood biomolecules with early rhythm control in atrial fibrillation patients: Exploratory analysis of the EAST-AFNET 4 Biomolecule Study

Christoph Al-Taie[1,2,3], MSc, Julius Obergassel[1,2,3], MD, Katrin Borof[2], MD, Julius Ridder[1,2,3], MSc, Andreas Rillig[2,3], MD, Andreas Metzner[2,3], MD, Andreas Goette[4,5,6], MD, Christina Magnussen[2, 3], MD, Moritz F Sinner[6,7,8], MD, MPH, Laura C. Sommerfeld[1,2,3], PhD, Stephan Willems[6,9], MD, Tanja Zeller[3,10], PhD, Renate B Schnabel[1,2,3,6], MD, Ulrich Schotten[6,11], MD, PhD, Antonia Zapf[3,6,12], PhD, Paulus Kirchhof[2,3, 6,13], MD, Larissa Fabritz[1,2,3,6,13], MD

(1) University Centre of Cardiovascular Science, University Medical Center Hamburg Eppendorf, Hamburg, Germany (2) Department of Cardiology, University Heart and Vascular Centre Hamburg (UHZ), Hamburg, Germany (3) German Centre for Cardiovascular Research (DZHK), Partner Site North, Martinistr. 52, Hamburg 20246, Germany (4) Saint Vincenz Hospital Paderborn, Department of Cardiology and Intensive Care Medicine, Paderborn, Germany (5) Medical Faculty, Otto-von-Guericke University, Magdeburg, Germany (6) Atrial Fibrillation NETwork (AFNET), Mendelstr. 11, 48149 Münster, Germany (7) Department of Medicine I, LMU University Hospital of Munich, Munich, Germany (8) German Centre for Cardiovascular Research (DZHK), Partner Site Munich Heart Alliance, Munich, Germany (9) Asklepios Clinic St. Georg, Cardiology and internal intensive care medicine , Hamburg, Germany (10) Institute for Cardiogenetics, UKSH, Lübeck, Germany (11) Maastricht University, Department of Physiology, Maastricht, Netherlands (The) (12) Institute of Medical Biometry and Epidemiology, University Medical Center Hamburg Eppendorf, Hamburg, Germany (13) Cardiovascular Sciences, University of Birmingham, B15 2TT Birmingham, UK

Short title: Biomarkers and Treatment Effect in EAST-AFNET 4

Keywords: Atrial fibrillation; Early rhythm control; Cardiovascular biomarkers; BMP10; Interaction analysis

Corresponding author:

Prof. Dr. Larissa Fabritz

University Centre of Cardiovascular Science & Department of Cardiology

University Heart and Vascular Center, University Medical Centre Hamburg Eppendorf

Martinistraße 52

20246 Hamburg

Phone: +49 (0)40 7410-57980

Email: l.fabritz@uke.de

Supplementary Tables

Supplemental Table 1: R packages including version numbers as used in the analysis scripts.

Package	Version
boot	1.3.31
broom.mixed	0.2.9.6
dplyr	1.1.4
ggplot2	3.5.2
glmnet	4.1.8
grid	4.2.2
gridExtra	2.3
lme4	1.1.36
mice	3.17.0
miceadds	3.17.44
mitools	2.4
openxlsx	4.2.8
readr	2.1.5
survival	3.7.0
survminer	0.5.0
tableone	0.13.2
tibble	3.2.1
tidyr	1.3.1
timeROC	0.4
writexl	1.5.1

Supplemental Table 2: The p-values for interaction for each biomolecule (continuously measured) and treatment effect. Integrating each biomolecule concentration without grouping of patients by quintiles of biomolecule concentrations.

Biomolecule	Unit	Range	*p* for interaction	*q* for interaction
**ANGPT2**	(ng/ml)	0.22 – 26.36	0.689	0.787
**BMP10**	(ng/ml)	1.05 – 4.96	0.164	0.787
**CA125**	(U/ml)	2.03 – 392	0.349	0.787
**CRP**	(mg/l)	0 – 447.56	0.499	0.787
**TnT**	(ng/l)	3 – 1084	0.550	0.787
**D-dimer**	(ng/ml)	0 – 6.34	0.066	0.787
**ESM1**	(ng/ml)	0.84 – 24.62	0.574	0.787
**FABP3**	(ng/ml)	11.44 – 25.06	0.900	0.900
**FGF23**	(pg/ml)	41.02 – 3901.60	0.576	0.787
**GDF15**	(pg/ml)	400 – 18255	0.731	0.787
**IGFBP7**	(ng/ml)	42.90 – 355.46	0.649	0.787
**IL-6**	(pg/ml)	1.5 – 1376	0.420	0.787
**NT-proBNP**	(pg/ml)	5.51 – 25147	0.617	0.787
**sCr**	(µmol/l)	36 – 577	0.609	0.787

Supplemental Table 3: Clinical characteristics stratified by BMP10 quintile groups and sex. AF: Atrial fibrillation, BMI: body mass index, BMP10: bone morphogenetic protein 10, FU Follow-up, EHRA: European Heart Rhythm Association, IQR: interquartile range, LA: left atrium, LVEF: left ventricular ejection fraction, NYHA: New York Heart Association.

	**BMP10 Low 1st quintile**		**BMP10 Intermediate 2nd – 4th quintile**		**BMP10 High 5th quintile**	
	**female**	**male**	**female**	**male**	**female**	**male**
**BMP10 in ng/ml (median [IQR])**	1.64 [1.54, 1.71]	1.60 [1.50, 1.70]	2.15 [1.97, 2.32]	2.07 [1.93, 2.24]	2.92 [2.69, 3.20]	2.87 [2.68, 3.17]
**n (patients)**	86	232	445	508	182	133
**Sinus rhythm at baseline**	67 (78%)	169 (73%)	297 (67%)	245 (48%)	77 (42%)	35 (26%)
**Sinus rhythm at FU 12 (%)**	68 (79%)	175 (75%)	346 (78%)	318 (63%)	107 (59%)	67 (50%)
**Sinus rhythm at FU 24 (%)**	62 (72%)	172 (74%)	313 (70%)	284 (56%)	101 (56%)	56 (42%)
**Treatment group: Early rhythm control**	47 (55%)	124 (53%)	217 (49%)	259 (51%)	91 (50%)	62 (47%)
**Sex: female**	86 (100%)	0 (0%)	445 (100%)	0 (0%)	182 (100%)	0 (0)
**Age (years)**	70 [64, 75]	69 [62, 73]	71 [66, 76]	70 [65, 75]	74 [69, 77]	73 [68, 78]
**BMI**	30 [27, 35]	30 [27, 32]	29 [25, 33]	29 [26, 32]	27 [24, 32]	28 [25, 31]
**Blood pressure (diastolic, mm Hg)**	80.28 (12.93)	80.90 (10.82)	81.03 (12.07)	82.08 (12.49)	82.01 (12.43)	80.33 (10.45)
**Blood pressure (systolic, mm Hg)**	135 [130, 150]	135 [124, 142]	137 [125, 150]	135 [122, 148]	139 [125, 145]	130 [120, 145]
**LVEF (%)**	60 [55, 65]	60 [55, 65]	61 [60, 65]	60 [53, 64]	60 [55, 65]	55 [45, 60]
**AF type (first episode)**	34 (40%)	86 (37%)	169 (38%)	157 (31%)	63 (35%)	51 (38%)
**AF type (paroxysmal)**	45 (52%)	100 (43%)	199 (45%)	166 (33%)	50 (28%)	30 (23%)
**AF type (persistent)**	7 (8%)	46 (20%)	77 (17%)	185 (36%)	69 (38%)	52 (39%)
**Other clinical characteristics**						
**Diabetes**	21 (24%)	69 (30%)	81 (18%)	142 (28%)	44 (24%)	39 (29%)
**Hypertension**	73 (85%)	187 (81%)	363 (82%)	403 (79%)	152 (84%)	94 (71%)
**Chronic kidney disease**	12 (14%)	14 (6%)	67 (15%)	47 (9%)	33 (18%)	22 (17%)

Estimated glomerular filtration rate (mL/min 1.73 m²)	78 [61, 91]	79 [69, 89]	74 [61, 85]	78 [66, 89]	70 [57, 79]	72 [58, 85]
Previous stroke or transient ischaemic attack	11 (13%)	33 (14%)	40 (9%)	76 (15%)	24 (13%)	11 (8%)
Chronic obstructive pulmonary disease	7 (8%)	13 (6%)	34 (8%)	38 (8%)	20 (11%)	12 (9%)
LA diameter (diastolic, mm)	42 (8)	45 (9)	41 (7)	46 (9)	42 (8)	44 (8)
NYHA class						
I	6 (7%)	16 (7%)	37 (8%)	66 (13%)	28 (15%)	17 (13%)
II	14 (16%)	53 (23%)	79 (18%)	100 (20%)	42 (23%)	36 (27%)
III	6 (7%)	12 (5%)	9 (2%)	15 (3%)	6 (3%)	12 (9%)
No heart failure	60 (70%)	151 (65%)	320 (72%)	327 (64%)	106 (58%)	68 (51%)
EHRA score						
I	19 (22%)	75 (32%)	112 (25%)	178 (35%)	39 (21%)	45 (34%)
II	37 (43%)	107 (46%)	215 (48%)	253 (50%)	91 (50%)	57 (43%)
III	24 (28%)	27 (12%)	87 (20%)	48 (9%)	33 (18%)	25 (19%)
IV	0 (0%)	5 (2%)	7 (1%)	2 (0.4%)	2 (1%)	1 (1%)
Missing	6 (7%)	18 (8%)	24 (5%)	27 (5%)	17 (9%)	5 (4%)
CHA_2DS_2-VASc score (mean (SD), median [IQR]))	4 (3), 3 [3, 5]	3 (1), 3 [2, 4]	4 (1), 3 [3, 4]	3 (1), 3 [2, 4]	4 (1), 4 [3, 5]	3 (1), 3 [2, 4]

Supplementary Figures

Supplemental Figure 1:

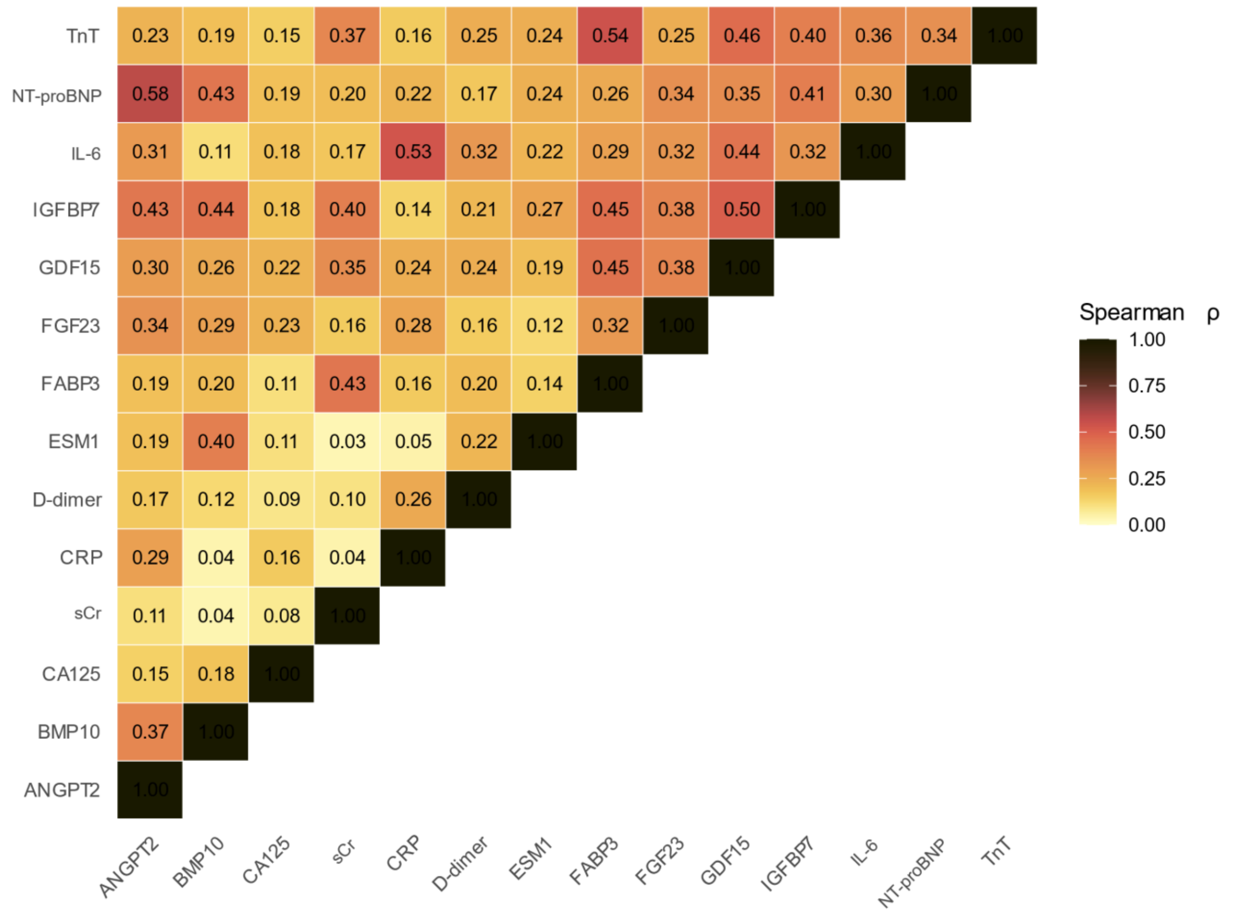

Supplemental Figure 1: Heatmap showing Spearman correlation ρ for all 14 quantified biomolecules from EAST-AFNET 4.

Supplemental Figure 2:

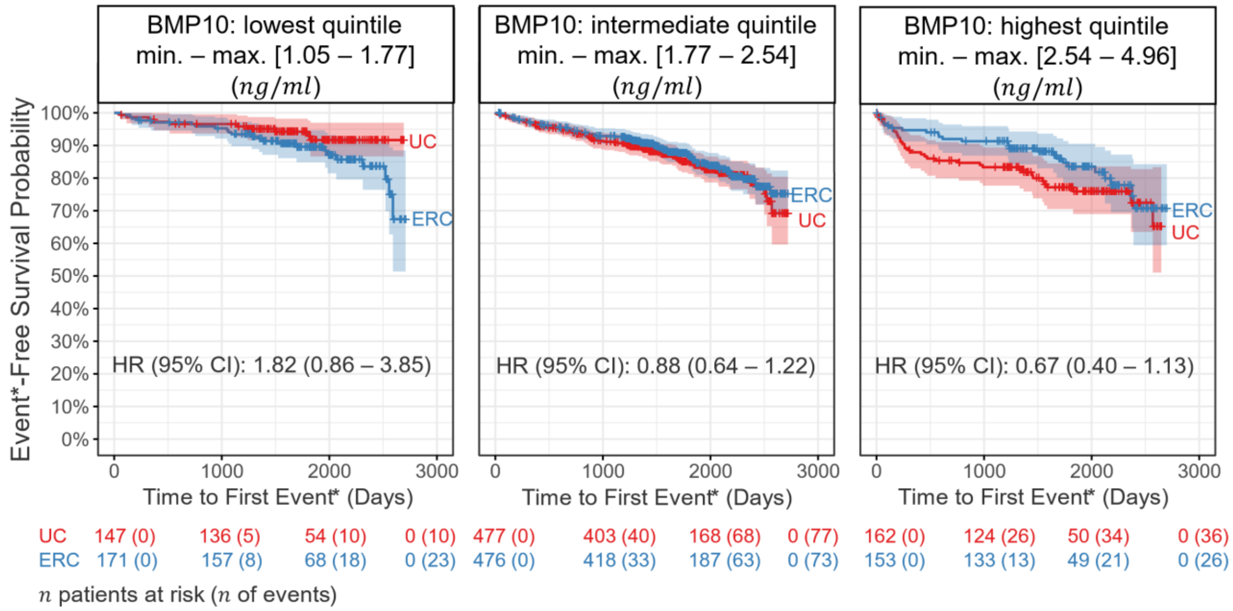

Supplemental Figure 2: Primary safety outcome and sensitivity analysis. Estimated Event-Free Survival Probability for the *primary safety outcome was a composite of death, stroke, or serious adverse events related to rhythm-control therapy. Header of each group shows BMP10 concentration in ng/ml as median and interquartile range (IQR) as a range from 25th to 75th percentile in the low, intermediate and high groups constructed by the discrete biomolecules according to their quintiles derived after natural log-transformation and 1% upper-winsorization.

Supplemental Figure 3:

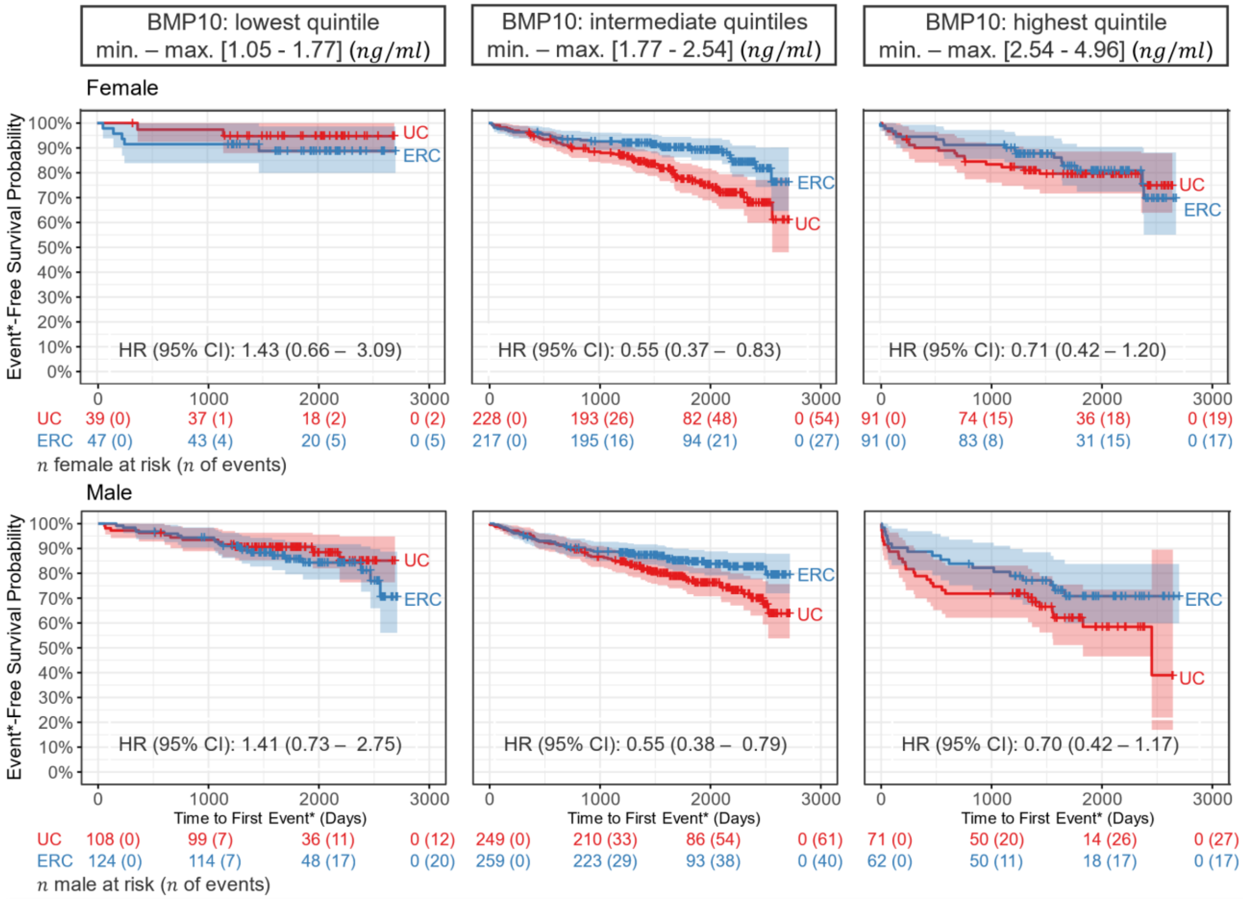

Supplemental Figure 3: Two-way interaction between sex and discretized bone morphogenetic protein 10 (BMP10) with the treatment group. ERC: early rhythm control, HR: hazard ratio, UC: usual care.

Supplemental Figure 4:

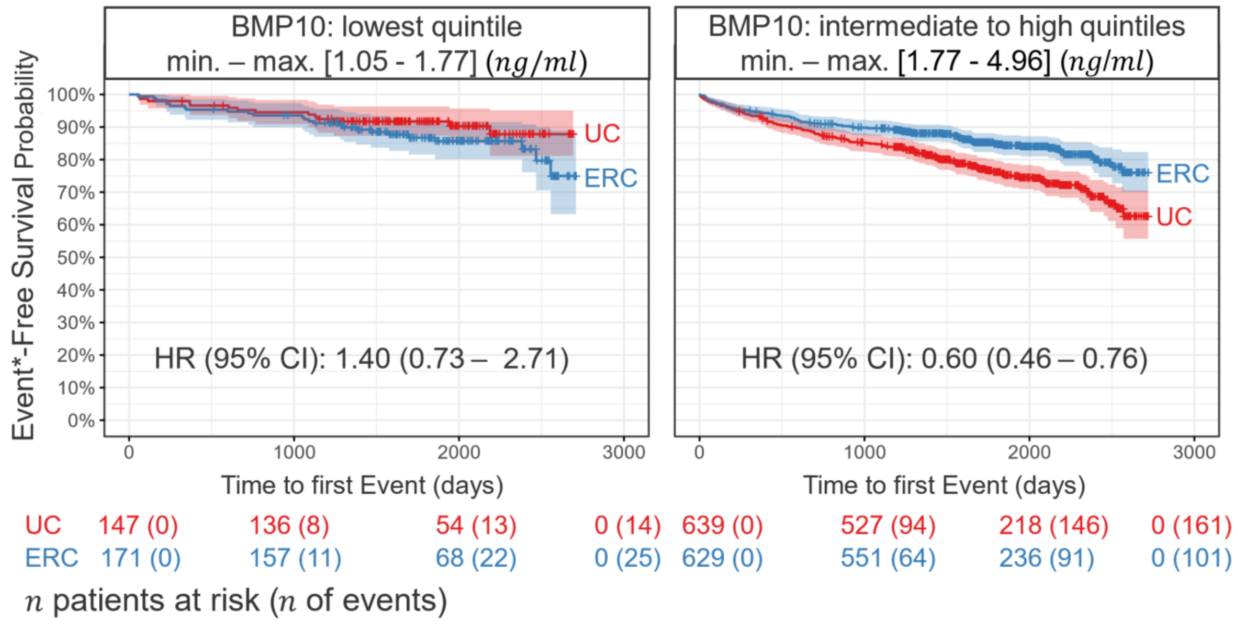

Supplemental Figure 4: Event free survival probability for the first primary outcome over the median follow-up time of 4.8 years stratified by treatment type aka random group. On the left for patients with bone morphogenetic protein 10 (BMP10) blood plasma concentrations measured at baseline in the first quintile and on the right for patients with protein levels in the second to fifth quintile. ERC: early rhythm control, HR: hazard ratio, UC: usual care.

Supplemental Figure 5:

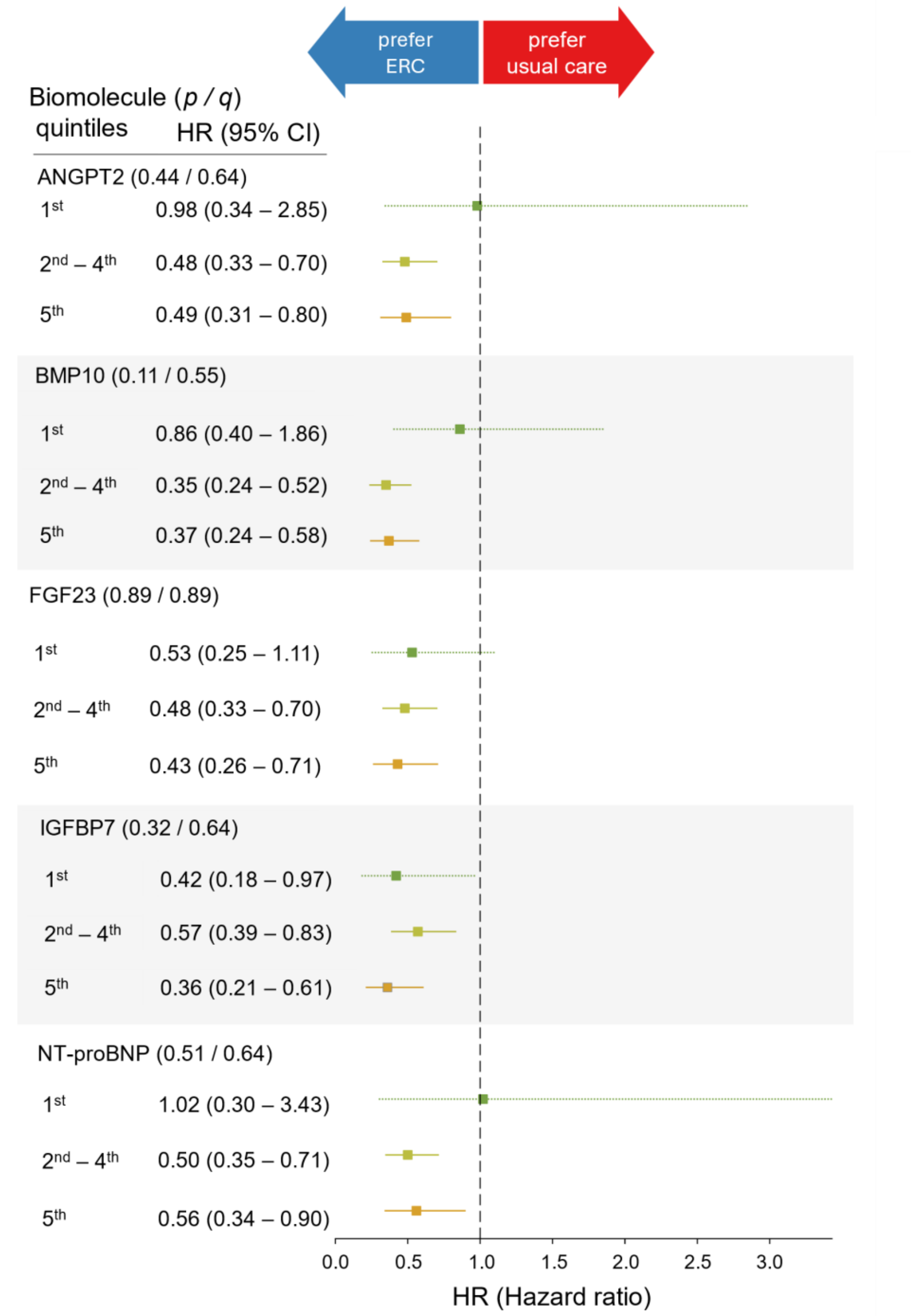

Supplemental Figure 5: Biomolecule interaction with early rhythm control therapy in the developed external replication cohort (BBC AF + TRUST snapshot). Hazard ratios of early rhythm control (vs usual care stemming from Cox proportional hazard (PH) models showing HRs alongside 95% CIs for the treatment) in interaction with each low, intermediate and high discretized biomolecule concentration group. The p-values for interaction were calculated using ANOVA comparing each nested Cox PH model pair. The q-values for interaction were calculated applying false discovery rate (FDR).
